# The Relationship Between Metabolic Syndrome and Psychological Resilience in Schizophrenia Patients in Remission

**DOI:** 10.1192/j.eurpsy.2025.2225

**Published:** 2025-08-26

**Authors:** Ö. Kaya Tuncel, D. İpekçioğlu, S. Uysal Atasoy

**Affiliations:** 1 Psychiatry, Republic of Turkey Ministry of Health Ordu State Hospital, Ordu; 2 Psychiatry, Bakırköy Mazhar Osman Mental Health and Neurological Diseases Education and Research Hospital, İstanbul, Türkiye

## Abstract

**Introduction:**

Comprehending metabolic syndrome (MetS) risk factors in schizophrenia patients is pivotal for devising effective treatment strategies.

**Objectives:**

This study aims to explore the relationship between MetS and psychological resilience in schizophrenia patients.

**Methods:**

A total of 140 schizophrenia patients with no hospital admissions or treatment changes in the last 6 months were enrolled in the study. For metabolic syndrome screening, patients’ blood pressure, height, weight, waist circumference, and hip circumference measurements were taken; these data, along with blood lipid levels and fasting glucose, were recorded on the sociodemographic and clinical data form. Patients were categorized into two groups based on the presence or absence of MetS. The Positive and Negative Syndrome Scale (PANSS) and the Resilience Scale for Adults were applied cross-sectionally.

**Results:**

MetS was identified in 33.6% of the included patients, with 88.6% exhibiting abdominal obesity. There were no statistically significant differences between groups in terms of gender, marital status, education level, employment status, type of antipsychotic, and use of single or multiple antipsychotics. In the MetS group, PANSS negative symptom scores were significantly higher. No significant differences were observed in psychological resilience between the groups.

**Image 1:**

**Figure:**
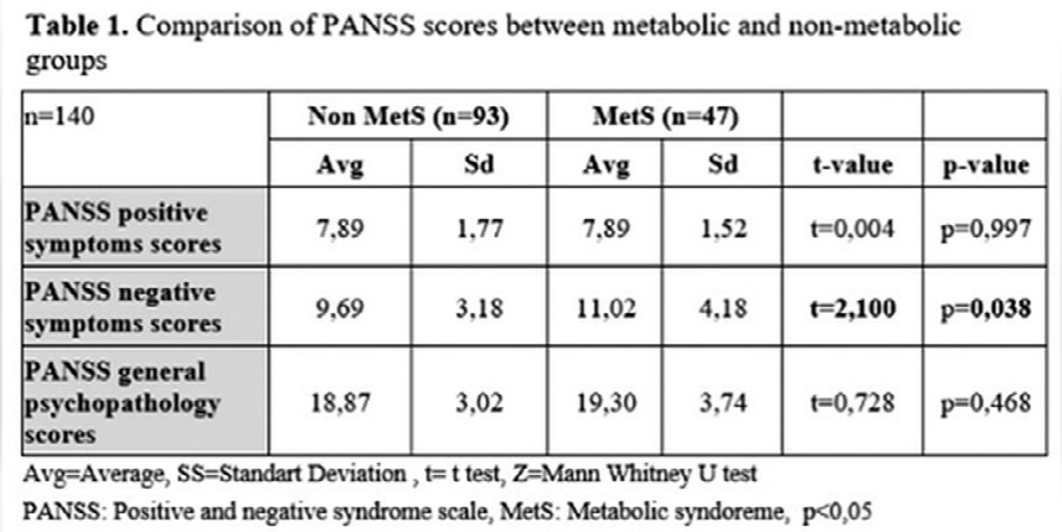

**Conclusions:**

The demonstration of the relationship between the presence of MetS and the severity of negative symptoms is one of the significant outcomes of our study. These outcomes may contribute to formulating personalized treatment approaches.

**Disclosure of Interest:**

None Declared

